# The perinatal health challenges of emerging and re-emerging infectious diseases: A narrative review

**DOI:** 10.3389/fpubh.2022.1039779

**Published:** 2023-01-05

**Authors:** Veronica N. E. Malange, Gitte Hedermann, Ulrik Lausten-Thomsen, Steen Hoffmann, Marianne Voldstedlund, Anna J. M. Aabakke, Anna K. Eltvedt, Jørgen S. Jensen, Morten Breindahl, Lone Krebs, Michael Christiansen, Paula L. Hedley

**Affiliations:** ^1^Department for Congenital Disorders, Statens Serum Institut, Copenhagen, Denmark; ^2^Department of Obstetrics and Gynecology, Copenhagen University Hospital Rigshospitalet, Copenhagen, Denmark; ^3^Department of Neonatology, Copenhagen University Hospital Rigshospitalet, Copenhagen, Denmark; ^4^Department of Bacteria, Parasites & Fungi, Statens Serum Institut, Copenhagen, Denmark; ^5^Data Integration and Analysis, Statens Serum Institut, Copenhagen, Denmark; ^6^Department of Obstetrics and Gynecology, Copenhagen University Hospital - North Zealand, Hillerød, Denmark; ^7^Department of Obstetrics and Gynecology, Copenhagen University Hospital - Holbæk, Holbæk, Denmark; ^8^Department of Clinical Medicine, University of Copenhagen, Copenhagen, Denmark; ^9^Global Health Unit, Department of Paediatric and Adolescent Medicine, Copenhagen University Hospital, Copenhagen, Denmark; ^10^Department of Obstetrics and Gynecology, Copenhagen University Hospital - Amager and Hvidovre, Copenhagen, Denmark; ^11^Department of Biomedical Science, University of Copenhagen, Copenhagen, Denmark; ^12^Brazen Bio, Los Angeles, CA, United States

**Keywords:** perinatal health, communicable disease, emerging infectious disease, re-emerging infectious disease, disease outbreak, public health surveillance

## Abstract

The world has seen numerous infectious disease outbreaks in the past decade. In many cases these outbreaks have had considerable perinatal health consequences including increased risk of preterm delivery (e.g., influenza, measles, and COVID-19), and the delivery of low birth weight or small for gestational age babies (e.g., influenza, COVID-19). Furthermore, severe perinatal outcomes including perinatal and infant death are a known consequence of multiple infectious diseases (e.g., Ebola virus disease, Zika virus disease, pertussis, and measles). In addition to vaccination during pregnancy (where possible), pregnant women, are provided some level of protection from the adverse effects of infection through community-level application of evidence-based transmission-control methods. This review demonstrates that it takes almost 2 years for the perinatal impacts of an infectious disease outbreak to be reported. However, many infectious disease outbreaks between 2010 and 2020 have no associated pregnancy data reported in the scientific literature, or pregnancy data is reported in the form of case-studies only. This lack of systematic data collection and reporting has a negative impact on our understanding of these diseases and the implications they may have for pregnant women and their unborn infants. Monitoring perinatal health is an essential aspect of national and global healthcare strategies as perinatal life has a critical impact on early life mortality as well as possible effects on later life health. The unpredictable nature of emerging infections and the potential for adverse perinatal outcomes necessitate that we thoroughly assess pregnancy and perinatal health implications of disease outbreaks and their public health interventions in tandem with outbreak response efforts. Disease surveillance programs should incorporate perinatal health monitoring and health systems around the world should endeavor to continuously collect perinatal health data in order to quickly update pregnancy care protocols as needed.

## 1. Introduction

Infectious diseases are caused by viral, bacterial, parasitic, or fungal pathogens that can be transmitted from one person to another (i.e., communicable diseases), or from vectors and/or other sources of contamination (1). Outbreaks occur when the number of disease cases increase above what would normally be expected in a given community, geographical area or season (2). Outbreaks may become epidemics (transmission within a particular geographical location) or pandemics (transmission across a large geographical region) (2). Emerging infectious diseases (EID) are those that have newly appeared in a population or are rapidly increasing in incidence or geographic range (3) e.g., Coronavirus disease 2019 (COVID-19), Middle East respiratory syndrome (MERS), Severe acute respiratory syndrome (SARS) and human immunodeficiency virus (HIV). Re-emerging infectious diseases are those that reappear after a previous decline in incidence (4) e.g., tuberculosis, poliovirus, measles, Ebola virus disease (EVD) and pertussis. Additionally, endemic diseases are those that occur at a constant level within a particular geographic region e.g., influenza and malaria, but which could be considered re-emerging in the event of a sudden increase in case numbers following a period of decline.

Communicable diseases together with maternal, neonatal, and nutritional diseases (CMNN) accounted for 26.4% of the worldwide total burden of disease in 2019, injuries accounted for 9.8% and non-communicable diseases made up the remaining 63.8% (5). The Sustainable Development Goal 3 (SDG-3), defined by the United Nations, is aligned with the aims of reducing the burden of disease and mortality caused by CMNN diseases. Consequently, many countries around the world are investing in innovations, interventions and monitoring systems which might reduce maternal mortality, end preventable deaths under 5 years of age, and fight communicable disease among other SDG-3 targets (6). However, burden of disease estimates demonstrate an unequal distribution with 60% of diseases in low-income countries and <5% in high-income countries categorized as CMNN (5). Furthermore, losses in disability-adjusted life years caused by CMNN diseases in South Asia and Sub-Saharan Africa is about 25 per 100 individuals with that of the Central African Republic exceeding 50 per 100, whereas that of Europe and North America is only 2, 5 per 100 individuals (5). While high income countries have been more successful at managing infectious diseases overall, the interconnectedness (mass international trade and travel) means that we are more likely to see cross-border transmission of EIDs than in the past, making the surveillance and control of infectious diseases and their consequences a global health issue.

**Figure 1:**
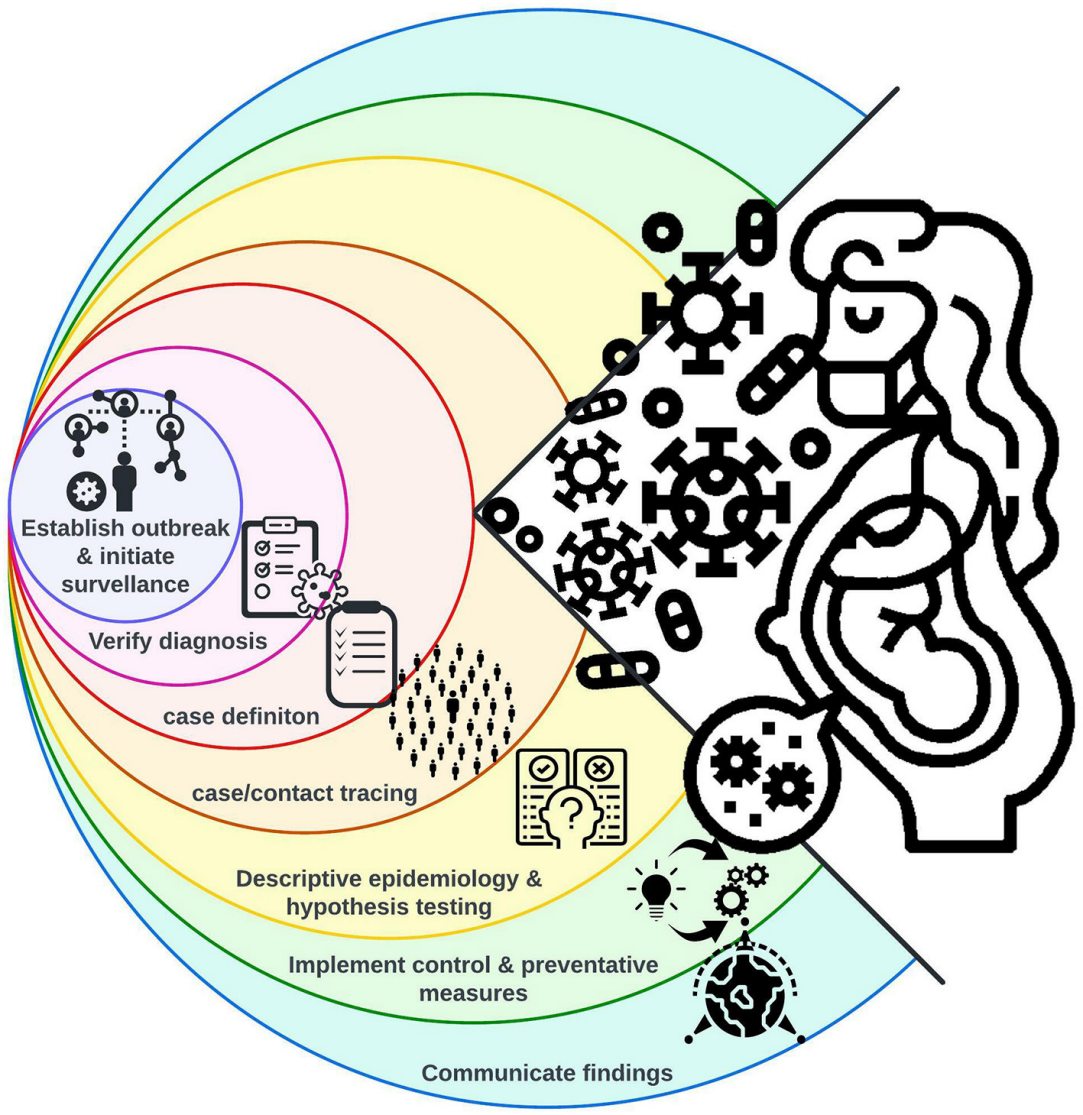
The nine epidemiological principles (condensed into seven steps) employed in outbreak response [adapted from (2)]. Consideration of the at-risk pregnant population should be built into each step allowing for continuous monitoring of perinatal health outcomes and rapid provision of necessary pregnancy care.

The epidemiological principles regarding detection and response to outbreaks of infectious diseases have been expertly presented in Houlihan and Whitworth 2019 (2) and are graphically summarized in Figure 1. Important infectious diseases are frequently monitored using global surveillance programs, although not all EIDs which caused outbreaks between 2010 and 2020 were monitored at a regional or global level (Table 1). Furthermore, the importance of zoonoses has driven a need to incorporate human, animal, and environmental health surveillance using the One Health approach (8), additionally, digital media has been employed to rapidly share and monitor outbreaks (9). Following, diagnosis verification and the creation of a case definition, contact tracing and descriptive epidemiology provides the foundation of the outbreak response and allows for the implementation of control and preventative measures (Figure 1). We argue that focused monitoring of the pregnant population should be added to the international outbreak response toolbox.

**Table 1:** Examples of long-term, continuous, regional, and global programs used for monitoring infectious diseases.

**Infectious disease**	**Regional or global monitoring programs**
Bourbon virus	-
Crimean-Congo hemorrhagic fever	EBO-SURSY Project - West and Central Africa
Chikungunya	-
Cholera	Global Task Force on Cholera Control (GTFCC)
*Colpodella spp*.	-
COVID-19	COVID-19 Sentinel Surveillance
Ebola	EVD Surveillance system
Guinea worm	-
Influenza	Global Influenza Surveillance and Response System (GISRS)
	European Influenza Sentinel Surveillance (EISS)
Lassa fever	-
Lyme disease	-
Measles	ECDC measles and rubella monitoring - Europe
	Surveillance for Vaccine Preventable Diseases
MERS	Regional Event Based Surveillance System
Mojiang Paramyxovirus	-
Monkeypox	-
Nipah virus	-
Ntwetwe virus	-
Pertussis	-
Plasmodium cynomolgi	-
Rat hepatitis E virus	-
Sosuga virus	-
Variegated Squirrel Bornavirus 1	-
Yellow fever	ECDC yellow fever surveillance - Europe
Zika	Zika Active Pregnancy Surveillance System (ZAPSS)

List of diseases adapted from (7).

During an infectious disease outbreak, the pregnant population is often exposed to additional risks compared to the non-pregnant population. They might be more susceptible to infection and/or at increased risk of severe disease due to the physiological changes of pregnancy (10). Furthermore, there's a risk of vertical transmission or adverse pregnancy outcomes. The pathological processes may have a profound effect on maternal and fetal health, particularly during the perinatal period (22 completed weeks of gestation to 7 days after birth) where placental abnormalities, intrauterine growth restrictions, stillbirth, preterm birth and congenital anomalies are common adverse pregnancy outcomes (11).

Generally, perinatal health outcomes are well described in re-emerging infectious diseases where significant pregnancy complications and adverse birth outcomes e.g., influenza and Zika virus disease, are known from reports of previous outbreaks. However, during EID outbreaks, where evidence of perinatal health outcomes has not been established, this information may be slow to emerge. Additionally, very few studies have described how the various public health interventions, beyond pharmaceutical interventions, implemented to curb outbreaks have affected perinatal health. In this narrative review we aim to summarize what is known about the impact of infectious diseases—and the interventions employed to control them—on perinatal health, focusing on several diseases which caused highly publicized outbreaks and epidemics in the past decade.

## 2. Methods

This narrative review was conducted in February 2021 to identify articles reporting perinatal health outcomes (outcome terms listed in Supplementary Table 1) with infectious disease outbreaks (exposure terms listed in Supplementary Table 1) in neonates (population term listed in Supplementary Table 1). MEDLINE, Embase, and Web of Science were searched using a combination of population, exposure, and outcome related search terms (listed in Supplementary Table 1). Limiting the search to English language studies published between, 1st January 2010 until the 17th of February 2021, of outbreaks between 2010 and 2020, resulted in the identification of 807 studies. Original studies (cohort, case-control, and cross-sectional studies) which investigated the perinatal health consequences of infectious disease outbreaks and their interventions were included. Review articles, correspondence, and editorials were excluded, ultimately 25 articles were included and form the base of this review (Supplementary Figure 1 and Supplementary Table 2). Additional studies were identified by examining the reference lists of relevant articles and by searching for relevant information produced outside of academic publishing practices (gray literature) using the google search engine. Each article was reviewed to determine the perinatal outcomes. As this search did not identify infectious disease outbreaks which had not been described specifically as such in the literature, an additional search of PubMed and Google scholar was performed to identify relevant literature for diseases not covered in the initial literature search. In this case the outcome search terms (Supplementary Table 1) were combined with the disease name (Table 2).

**Table 2:** The hallmarks of the perinatal impacts of the described emerging and re-emerging infectious diseases.

**Disease**	**Mode of transmission**	**Hallmarks of the perinatal outcomes**
Influenza virus disease	Droplets (coughing and sneezing), close contact.	PTB; SGA; Low Apgar score at birth.
Ebola virus disease	Direct contact with body fluids (blood, vomit, breastmilk, semen, sweat, urine and stool).	Maternal death; fetal loss; Pregnancy-associated hemorrhages; stillbirth; spontaneous abortions; neonatal death.
Measles disease	Direct contact with infectious droplets, airborne (breathes, sneezes, coughs).	Spontaneous abortions; PTB; fetal death; congenital infections.
Pertussis disease	Close contact, respiratory secretions (coughing and sneezing)	Neonatal death
Zika virus disease	vector transmission (mosquito: Aedes species), blood borne, sexual transmission	Microcephaly; Congenital malformations.
MERS-CoV disease	Close contact, respiratory secretions (coughing), animal-to-human transmission (camels)	Maternal death; PTB; stillbirth.
COVID-19 disease	Close contact, droplet, airborne, fomite, fecal-oral, blood-borne, mother-to-child, and animal-to-human transmission	PTB; Stillbirth; LBW, SGA

PTB, Preterm Births; LBW, Low Birth Weight; SGA, Small for Gestational Age.

The time from the declaration of an outbreak to the first report of evidence-based perinatal health implications regarding that outbreak was assessed by searching PubMed for articles containing both the outbreak disease (listed in Table 1) and perinatal health outcome search terms (listed in Supplementary Table 1) in either the title or the abstract. Only cohort studies or case/control studies were included as case reports and case series, often the first study types reported during an EID outbreak, may not provide sufficient strength of evidence to produce generalizable results.

## 3. Results and discussion

A total of 25 articles were identified which related to infectious disease outbreaks and perinatal health (Supplementary Table 2). In terms of article type, 21 cohort studies, three cross-sectional studies, and one case-control study were identified. The articles were predominantly focused on the implications of influenza infection (16%) and influenza vaccination (64%) in pregnancy, particularly studies related to the 2009–2010 H1N1 pandemic (52%). Studies of pertussis vaccination (8%) and hospitalization (4%), SARS-CoV2 infection (4%) and COVID-19 lockdown conditions (4%) were also identified.

### 3.1. Global infectious disease outbreak trends in the last decade

Over the past decade, numerous infectious disease outbreaks have occurred globally, mostly as epidemics, but pandemics have also been reported. Most of these infectious diseases are re-emerging (e.g., influenza and EVD) but we have also seen the appearance of EIDs like MERS and COVID-19 which pose challenges to global public and perinatal health.

Some of the most widely reported cross-border outbreaks occurring between 2010 and 2020 include influenza (H1N1) (2009–2010), MERS (2012 until present), EVD (2014 to 2016, and 2018 until present), Zika virus disease (2015–2016), and COVID-19 (2019 to present). Additionally, smaller outbreaks of re-emerging diseases e.g., measles, and pertussis have been reported in many countries around the world, which likely occur because of vaccine hesitancy (e.g., measles) (12, 13) and waning immunity (e.g., pertussis) (14) (Table 2).

There are two categories of public health interventions employed to mitigate and control infectious disease outbreaks: pharmaceutical interventions such as medications and vaccines and non-pharmaceutical interventions (NPIs). NPIs include personal protective measures (e.g., wearing facemasks, hand hygiene measures, using mosquito nets, practicing safe sex, etc.), environmental measures (e.g., surface and object cleaning, using mosquito repellents, etc.), physical distancing measures (e.g., quarantine, limiting the number of persons in public areas and work from home policies), and travel-related measure (e.g., entry and exit screening, and border closures). NPIs help to reduce the disease transmission and number of cases in the population, which is important for protecting vulnerable members of the society, including pregnant women. However, NPIs, particularly physical distancing measures, may present challenges to pregnant women should they interfere with routine prenatal care. Ideally, NPIs should be employed in such a manner as to support access to care while also allaying the fears pregnant women may have when seeking routine prenatal care during an infectious disease outbreak. Pharmaceutical interventions may also present challenges, particularly for treating EIDs, as safety of use in pregnancy must be established.

### 3.2. Impacts of several widely reported infectious disease outbreaks, which occurred between 2010 and 2020, on perinatal health

#### 3.2.1. Re-emerging diseases

##### 3.2.1.1. Influenza outbreaks and perinatal health

Seasonal epidemics of human influenza A and B virus infection occur almost yearly (winter seasons) in temperate locations, hence it is referred to as “seasonal” influenza while in tropical regions, influenza may occur all through the year, causing irregular outbreaks (15). When outbreaks of re-emerging, endemic diseases like influenza occur they may cause epidemic or pandemic disease.

Influenza infection in pregnant women increases the likelihood of preterm birth and delivering small for gestational age (SGA) infants (16–18). During the 2009–2010 swine flu pandemic, infants born to women infected with the H1N1 strain were more likely to be born preterm, SGA, and have a low Apgar score (18). However, while the first cases of H1N1 infection in pregnant women were published a month after the outbreak was reported (19), the first paper describing the perinatal consequences of H1N1 infection during pregnancy was first published 11 months after the initial outbreak was reported (Table 3) (20).

**Table 3:** Time of establishing perinatal consequences (first cohort or case-control report) from the start of a disease outbreak (declaration date or month of first cases) for emerging disease outbreaks since 2010.

**Emerging infection/disease outbreak**	**Start date**	**Time to establishment of perinatal consequences**	**References**
Swine flu (H1N1) (pandemic)	25 Apr 2009	11 months	(20)
Cholera (Haiti)	20 Oct 2010	22 months	(21)
*Plasmodium cynomolgi* (Malaysia)	Jan 2011	–	
Swine flu (H3N2v) (USA)	Jul 2011	–	
Lassa fever (Ghana)	Oct 2011	–	
Mojiang paramyxovirus (China)	Jun 2012	–	
Sosuga virus (Uganda)	Aug 2012	–	
MERS-CoV disease (Middle East)	Sep 2012	–	
Avian flu (H7N9) (China)	Mar 2013	40 months	(22)
*Colpodella* sp. (Heilongjiang-China)	May 2013	–	
Variegated squirrel bornavirus 1 (Germany)	Jun 2013	–	
Chikungunya (Caribbean)	Dec 2013	–	
Ebola virus disease (West Africa)	8 Aug 2014	40 months	(23)
Avian flu (H5N6) (China)	2014	–	
Lassa fever (Benin)	2014	–	
Bourbon virus (USA)	2014	–	
Zika virus disease (the Americas)	Aug 2015	5 months	(24)
CCHF (Spain)	2016	–	
Chikungunya (Pakistan)	Feb 2016	38 months	(25)
Lassa fever (Togo)	2016	–	
Ntwetwe virus (Uganda)	2016	–	
Monkeypox (Nigeria)	2017	–	
Yellow fever (Brazil)	2017	–	
Rat hepatitis E virus	2017	–	
Guinea worm (Angola)	2018	–	
Lyme disease (Nepal)	2018	–	
Avian flu (H7N4)	2018	–	
Monkeypox (Liberia, UK)	2018	–	
Nipah virus (India)	2018	–	
Ebola virus disease (DRC)	17 Jul 2019	–	
COVID-19 disease (pandemic)	30 Jan 2020	5 months	(26, 27)

List of diseases adapted from (7).

As much is known about influenza, public health responses including surveillance programs (Table 1), vaccination programs and modes of treatment are well established. Even though some new or particularly serious strains of influenza may sometimes occur, the public health response—built on preparedness programs—typically follows the same approach and focuses on increasing vaccination uptake among vulnerable groups including pregnant women (28–32). Nevertheless, vaccine hesitancy is a frequent challenge in mitigating influenza epidemics (33).

A study of pregnant Japanese women showed that vaccination reduced the rate of influenza infection by 89% and led to zero maternal deaths (34). On the whole, studies examining the effects of influenza vaccination during pregnancy found either no association (35–39) or a modest negative association with preterm birth (30–32, 40, 41). Indeed, a systematic review examining the safety of inactivated influenza vaccines found them to be safe with protective effects on preterm birth and low birth weight (LBW) (42).

Antiviral treatment is another pharmaceutical intervention recommended for pregnant women and post-partum women with suspected or confirmed influenza infection (28). This is mainly recommended by the regulatory agencies in the US (Centers for Disease Control and Prevention) and Europe. Used either prophylactically or empirically (usually within 48 h), antiviral treatment prevents severe disease, reduces admissions to the intensive care unit, reduces the risk of adverse pregnancy outcomes and maternal death (34, 43–45). A multinational study carried out in Denmark, Norway, Sweden, and France showed that the use of antiviral treatment was not associated with increased risk of adverse neonatal outcomes or congenital malformation (46), hence safe to use.

Much is known about influenza and its effect on perinatal health. There are several, effective and safe to use in pregnancy, pharmaceutical interventions at our disposal. While additional NPIs are needed to increase vaccination uptake among pregnant women [e.g., a digital intervention addressing beliefs about influenza infection and vaccination (47)] on the whole influenza surveillance and public health responses serve pregnant women well largely because the risks are well understood, consequently their needs are in focus during influenza outbreaks.

##### 3.2.1.2. Ebola virus disease outbreaks and perinatal health

Since 1976 there have been several EVD outbreaks, with the largest recorded epidemic being the 2014–2016 outbreak, affecting three continents (Africa, Europe, and North America) with most of the cases reported in West Africa. Following the declaration of this outbreak in March 2014, it took 40 months before the first publication addressed the perinatal health complications associated with this EVD outbreak (Table 3) (23). While pregnant women are not more susceptible to EVD (48), they are thought to be prone to severe illness and death if infected. Previous EVD outbreaks showed high maternal mortality rates from 74 to 100% whereas the 2014 EVD outbreak demonstrated reduced maternal mortality ranging between 39 and 42% (23, 49). Furthermore, pregnant women during the 2014 outbreak did not experience greater mortality than non-pregnant women (23). It must be noted that generally the 2014 EVD epidemic appears to have been less fatal than previous outbreaks probably due to small sample sizes and reporting bias (23, 49).

Ebola can be transmitted from mother to child either *in utero* (*via* hematogenous spread through the placenta to the fetal tissue and amniotic fluid), during delivery, or post-partum mainly from breast-feeding (49). An increased risk of pregnancy-associated hemorrhage and fetal loss has been reported for pregnant women with EVD (48, 50). Other neonatal outcomes associated with EVD in pregnancy include spontaneous abortions and stillbirths in almost 80% of the cases (49). Furthermore, 20% of the liveborn neonates died within 19 days of birth (50–56). However, recent evidence has shown that neonates born with congenital EVD can survive if treated with an experimental “cocktail” containing monoclonal antibodies (ZMapp), antiviral GS-5734, and a buffy coat transfusion from an Ebola survivor (57).

Vaccination with the vaccine rVSV-ZEBOV (ERVEBO^®^) is highly effective in preventing infection among exposed individuals above 6 years of age (58) and healthcare professionals. In 2020, the WHO recommended that pregnant and breastfeeding women, living in EVD affected areas, should be offered ERVEBO^®^ during an active EVD outbreak (59). NPIs such as isolation, contact tracing and travel restrictions during EVD outbreaks have also played a role to significantly curb the spread of the disease, but the impact of these measures on the perinatal health of uninfected women is unknown.

The development of ERVEBO^®^, and the recommendation that the vaccine can be offered to pregnant and breastfeeding women allows for better care provision of pregnant women living in EVD outbreak areas. However, the effects of the vaccine on pregnancy need to be evaluated and the delay of over 3 years between the 2014 EVD outbreak being declared and the publication of a cohort study examining the implications of EVD in pregnancy (23) (Table 3) support the need for adding focused monitoring of the pregnant population to outbreak response toolboxes globally.

##### 3.2.1.3. Measles outbreaks and perinatal health

Measles, primarily a disease of childhood, is another common re-emerging infectious disease that can lead to serious perinatal health issues. Vaccination is the primary public health intervention used to prevent measles infections, decrease disease severity, and reduce transmission in the general population. In many countries the vaccine requirement was increased from one to two doses in the late 1980s (60, 61), which provides life-long immunity. Consequently, national vaccination programs ensure that the perinatal impact of measles outbreaks is generally small. However, vaccine hesitancy has resulted in re-emergence of measles in many populations (13), potentially putting unvaccinated pregnant women and their fetuses at risk. Furthermore, women vaccinated before the adoption of the two-dose schedule may not have sufficient immunity and may therefore be more vulnerable during pregnancy.

When an unimmunized pregnant woman becomes infected with measles before fetal viability (Gestational Age, GA <22 weeks), the virus alters the body's immune tolerance to the fetus. Consequently, the fetus is perceived as foreign, which increases the likelihood of spontaneous abortion (62). Additionally, studies have shown that measles infection is associated with preterm delivery, fetal death and in some rare cases, congenital infections (63, 64). During a recent measles outbreak in Italy (2017–2018) 25% of infected pregnant women delivered prematurely (62).

Given that the vaccine is a live attenuated vaccine, it is contraindicated during pregnancy but can be administered before pregnancy or post-partum if the woman did not receive a vaccine in childhood or has had a primary infection. In the event of exposure to the measles virus, immunoglobulins can be given to pregnant women or neonates (within 6 days of exposure) to reduce the severity of the disease (63).

Since measles vaccinations cannot be safely administered to pregnant women, and the consequences of infection are potentially dire, public health initiatives relating to measles outbreaks should include a focus on preventing measles infection during pregnancy.

##### 3.2.1.4. Pertussis outbreaks and perinatal health

Even though vaccination coverage against pertussis (whooping cough) infection is one of the best worldwide, there have been several outbreaks in the last decade. This is because—since immunity wanes over time—adolescent and adult family members are likely reservoirs of the disease, thereby infecting susceptible newborns and infants before completion of their childhood vaccination (65). Furthermore, neonates have a higher risk of severe complications and death than older infants (66). Vaccination of pregnant women in their third trimester was first implemented in the USA following the 2011 pertussis outbreak (67), and has since been implemented in many other countries as the main pharmaceutical intervention to prevent neonatal pertussis (68). Systematic reviews have shown that maternal pertussis vaccination during pregnancy is safe and not associated with increased risk of any serious perinatal complications such as hypertensive disorder, preterm birth, stillbirth, neonatal death etc., (69, 70).

Vaccination against pertussis during the third trimester of pregnancy is a safe and effective method of preventing severe disease in neonates and infants. Pertussis surveillance and outbreak response is an excellent example where pregnancy care is in particular focus and an integral part of the outbreak response.

#### 3.2.2. Emerging diseases

##### 3.2.2.1. Zika virus outbreaks and perinatal health

Several outbreaks of Zika virus disease have occurred throughout the world with the most prominent one occurring between 2015 and 2016 affecting over 50 countries across the world (71). Zika virus disease presents with very mild symptoms in the mother and is indeed asymptomatic in most cases for both mother and child. However, its impact on perinatal health, especially in neonates—even if rarely occurring—is quite alarming. This is because Zika virus infection in pregnancy may cause congenital malformation such as microcephaly in babies. It took ~5 months for the Brazil Ministry of Health to determine the association between microcephaly and other congenital malformations, seen in increasing numbers at the time of the Zika virus outbreak (Table 3) (24). A systematic review and meta-analysis of 21 cohort studies of 35,568 pregnant women infected with Zika virus showed that the prevalence rate of congenital microcephaly was 3%. The prevalence rates of other adverse perinatal outcomes were 4% for fetal loss, 4% for SGA, 5% for LBW, and 7% for prematurity (72).

Several NPIs were used to reduce Zika transmission, including prevention of mosquito bites (using mosquito nets and repellents, and wearing long sleeves), avoiding unprotected sex with those infected by the virus, using condoms to prevent unwanted pregnancies and avoiding traveling to affected areas. In 2019, the WHO recommended that couples, living in an affected area, planning to conceive should consider delaying conception until the risk of infection had decreased (73). These measures helped to curb the infection in pregnant women and their babies. There is currently no specific treatment or vaccine available for Zika virus (74).

As an emerging disease the connection between Zika virus infection and perinatal health outcomes took 5 months to be established, leading to the public health bodies recommending couple to delay getting pregnant in affected areas. The monitoring of pregnancy and perinatal health data, whether at local, regional, or national levels should be prioritized, for the sake of providing adequate pregnancy care in the first instance, but such data could also be used to better inform outbreak monitoring and response initiatives.

##### 3.2.2.2. MERS-CoV outbreaks and perinatal health

Middle East Respiratory Syndrome Coronavirus (MERS-CoV) is a type of coronavirus, which causes severe acute respiratory illness and is associated with a high mortality rate. It was first discovered in 2012, predominantly in the Middle East countries with Saudi Arabia being the most affected country (75). The infection extended however to other parts of Asia, Africa, Europe, and the US. Possibly due to the low number of MERS cases, it took more than 2 years from when the outbreak started until the first article on perinatal outcomes was published, and only three pregnancies were examined (76). To date over 2,500 MERS cases have been reported in 27 countries (77), nearly a third of these would be expected to be female (78). Consequently, very few studies have been done regarding perinatal health and MERS-CoV; the main perinatal outcomes observed are stillbirth, preterm delivery, and maternal death (76, 79).

The main NPIs used to prevent MERS-CoV infections include personal protective measures (face mask, hand hygiene, etc.,), environmental measures (surface and object cleaning), and travel-related measures (travel advice, entry and exit screening, border closures). So far there is no vaccine to prevent MERS-CoV infection and the treatment is mainly symptomatic.

Due to a strong sex bias in MERS-CoV cases it is not surprising that there are no cohort or case-control studies reporting the implications of MERS-CoV infection for perinatal health, we can only emphasize on the need for further real-time monitoring of this population during future MERS-CoV outbreaks.

##### 3.2.2.3. COVID-19 pandemic and perinatal health

COVID-19 is the disease caused by severe acute respiratory syndrome coronavirus 2 (SARS-CoV2). This ongoing pandemic emerged in 2019 and was declared a pandemic in March 2020. Data on the characteristics of COVID-19, its presentation, and impact on pregnant women and perinatal outcomes were first published 5 months after the outbreak was discovered in China (Table 3) (26, 27). Pregnant women are at risk of developing severe disease and pregnancy complications because of coronavirus infections (e.g., SARS and MERS) and COVID-19 presents the same challenges (80). Furthermore, there is evidence of mother-to-child transmission, but neonatal COVID-19 through breastfeeding is rare (80). Systematic reviews of neonatal outcomes of children born to mothers with COVID-19 disease in pregnancy, have showed an increase in preterm birth rates, LBW and SGA (81–84). However, it is still unclear if these adverse events are due to the SARS-CoV-2 infection, or a result of other pregnancy complications worsened by the COVID-19 disease. It is important to note that the severity of risk will be affected by the population studied (85) and the severity of outcomes reported will be affected by the testing strategy employed for that population during the period under investigation (86), making it difficult to compare studies. This population-based difference in outcome severity can be seen clearly when comparing the initial case reports of COVID-19 in pregnancy (naturally studies of more severe, hospital-admitted cases) with later reports of population-based studies. Furthermore, secondary bacterial infections associated with COVID-19 infection e.g., Haemophilus influenzae type B (Hib) are strong predictors of poor outcomes (87). Pregnant women are at particularly high risk of Hib infection which is associated with adverse pregnancy outcomes (88).

Many NPIs were used to reduce SARS-CoV2 transmission, including personal protective measures (face mask, hand hygiene, etc.,), environmental measures (surface and object cleaning), physical distancing measures (quarantine, cordons sanitaire, isolation, contact tracing, etc.,), travel-related measures (travel advice, entry and exit screening, border closures) and community screening. NPIs such as quarantine and lockdown affect perinatal health both positively and negatively. For example, a prospective observational study in Nepal showed that perinatal outcomes like neonatal mortality, stillbirths, and extremely preterm births increased during the lockdown period compared to pre-lockdown period (89). According to the authors, this was probably due to psychosocial stress related to social restrictions. However, several other studies and independent reports from high-income countries like Canada, Denmark, and Ireland have shown that the rate of extremely preterm birth (<28 weeks) or very low birth weight deliveries was reduced during the COVID 19 lockdown period (90–95). The authors of the Danish studies speculated that the fall in extremely preterm birth rates may have been due to reduced anxiety, and/or decreased levels of physical activities in pregnant women during the lockdown period (92). They further posited that lower rates of other infections, that might trigger preterm labor, might partially explain the reduction in preterm births (92). The lockdown period in China has also been associated with an increase in the rates of cesarean delivery and in birth weight (96) presumably due to reduced physical activity during this period. The differences in perinatal health implications to COVID-19 NPIs may reflect the differences in how these NPIs were applied between countries as well as the impacts the NPIs themselves had on national resources and access to pregnancy care facilities.

Currently, vaccination is the main pharmaceutical intervention used to reduce the burden of COVID-19 disease on communities. Additionally, several pharmaceutical products can be used to treat severely ill people—including pregnant women, as they have shown very little or no fetal toxicity. They include antiviral agents such as lopinavir/ritonavir and remdesivir (97). However, attitudes toward vaccination among pregnant women vary between countries and regions (98). Vaccine hesitancy broadly related to concerns around vaccine safety, necessity, or efficacy (98, 99). However, hesitancy regarding COVID-19 vaccination in pregnancy and the community at large is a major barrier in achieving acceptable levels of protection for pregnant women and other vulnerable groups.

It took 5 months for the connection between SARS-CoV2 infection and perinatal health to be established (26, 27) and nearly 7 months for the impact COVID-19 mitigation measures were having on pregnancy and perinatal health to be reported in the literature (92). Indeed, monitoring of routinely collected pregnancy and perinatal health data allowed for the consequences of the COVID-19 NPIs to be evaluated at a population level. Including a specific focus on pregnancy care and perinatal health as a part of outbreak surveillance and response would allow real-time perinatal health monitoring and ensure the pregnant population is appropriately cared for during the next pandemic.

## 4. Conclusion

Monitoring perinatal health is an essential aspect of national and global healthcare strategies. This is because, this period of life has a critical impact on early life mortality as well as neurological, mental, and chronic health conditions later in life. For example, both SGA and large for gestational age babies are more likely to develop cardiometabolic diseases as adults than babies born with an appropriate birth weight for gestational age (100). Similarly, preterm delivery increases the risk of neurological and mental disabilities, and is responsible for causing 35% of neonatal deaths globally (101). Overall, we have achieved a lot in terms of reducing the global neonatal mortality rates from 30 to 17 deaths per 1,000 live births, between 2000 and 2019 (102). However, we are still some way from attaining SDG-3, which aims globally to reduce neonatal mortality rates to 12 deaths per 1,000 live births (6).

The unpredictable nature of emerging infections and the potential for adverse pregnancy and birth outcomes necessitates that we thoroughly assess pregnancy and perinatal health implications of disease outbreaks and their public health interventions in tandem with outbreak response efforts. Our literature review demonstrates that, of the 31 outbreaks investigated, only seven had reported the perinatal consequence of acquiring the disease while pregnant during the outbreak (Table 3). The median time to publication of perinatal health findings was 23 months (interquartile range (IQR) 8–39 months).

Considering the consequences of infectious diseases during pregnancy, we propose a policy of continuous collection of perinatal health data, from routinely collected medical data, in dedicated registers, managed by national or regional health authorities, as appropriate. Such a register would enable ongoing surveillance of perinatal health outcomes. Importantly, during infectious disease outbreaks epidemiological and clinical data from the register would enable rapid reporting of the perinatal impacts of the outbreak. This would improve confidence in the population and ensure threats to perinatal health are quickly identified, and provide the necessary information for implementing preventative or therapeutic care strategies.

Furthermore, an examination of historical outbreaks is necessary to determine best practices for addressing future emerging disease outbreaks.

## Author contributions

VM, MC, and PH informed the study protocol and conceptualized the project. VM and PH led on writing the manuscript with input from all co-authors and carried out the database search with input from GH and MC. UL-T, MC, and PH provided overall supervision, leadership, and advice. All authors reviewed and approved the final version of the manuscript.

## References

[1] van Seventer JM Hochberg NS. Principles of infectious diseases: transmission, diagnosis, prevention, and control. Int Encyclopedia Public Health. (2017) 22–39. 10.1016/B978-0-12-803678-5.00516-6

[2] Houlihan CF Whitworth JA. Outbreak science: recent progress in the detection and response to outbreaks of infectious diseases. Clin Med. (2019) 19:140–4. 10.7861/clinmedicine.19-2-140 30872298 PMC6454359

[3] Morse SS. Factors in the emergence of infectious diseases. In:Price-Smith AT, editor. Plagues and Politics. London: Palgrave Macmillan (1995). p. 8–26.

[4] Quaglio G Demotes-Mainard J Loddenkemper R. Emerging and re-emerging infectious diseases: a continuous challenge for Europe. Eur Respir J. (2012) 40:1312–4. 10.1183/09031936.00111712 23204016

[5] Roser M Ritchie H. Burden of disease. Our World in Data. (2016).

[6] WHO. Targets of Sustainable Development Goal 3. (2022). Available online at: https://www.euro.who.int/en/health-topics/health-policy/sustainable-development-goals/sustainable-development-goals-sdgs/targets-of-sustainable-development-goal-3 (accessed August 11, 2022).

[7] England PH. *Guidance: Emerging Infections: How Why they Arise*. London, UK: gov.uk (2019). Emerging infections: characteristics, epidemiology and global distribution [2019/02/27]. Available online at: https://www.gov.uk/government/publications/emerging-infections-characteristics-epidemiology-and-global-distribution/emerging-infections-how-and-why-they-arise (accessed March 25, 2022).

[8] Hasler B Cornelsen L Bennani H Rushton J A. review of the metrics for One Health benefits. Rev Sci Tech. (2014) 33:453–64. 10.20506/rst.33.2.2294 25707176

[9] Quinn E Hsiao KH Maitland-Scott I Gomez M Baysari MT Najjar Z. Web-based apps for responding to acute infectious disease outbreaks in the community: systematic review. JMIR Public Health Surveill. (2021) 7:e24330. 10.2196/24330 33881406 PMC8100883

[10] Sappenfield E Jamieson DJ Kourtis AP. Pregnancy and susceptibility to infectious diseases. Infect Dis Obstet Gynecol. (2013) 2013:752852. 10.1155/2013/752852 23935259 PMC3723080

[11] European Perinatal Health Report. Babies' Health: Mortality and Morbidity During Pregnancy and in the First Year of Life. Paris (2015).

[12] Majumder MS Cohn EL Mekaru SR Huston JE Brownstein JS. Substandard vaccination compliance and the 2015 measles outbreak. JAMA Pediatr. (2015) 169:494–5. 10.1001/jamapediatrics.2015.0384 25774618 PMC4476536

[13] Phadke VK Bednarczyk RA Salmon DA Omer SB. Association between vaccine refusal and vaccine-preventable diseases in the United States: a review of measles and pertussis. JAMA. (2016) 315:1149–58. 10.1001/jama.2016.1353 26978210 PMC5007135

[14] Chit A Zivaripiran H Shin T Lee JKH Tomovici A Macina D. Acellular pertussis vaccines effectiveness over time: a systematic review, meta-analysis and modeling study. PLoS ONE. (2018) 13:e0197970. 10.1371/journal.pone.0197970 29912887 PMC6005504

[15] Tamerius JD Shaman J Alonso WJ Bloom-Feshbach K Uejio CK Comrie A. Environmental predictors of seasonal influenza epidemics across temperate and tropical climates. PLoS Pathog. (2013) 9:e1003194. 10.1371/journal.ppat.1003194 23505366 PMC3591336

[16] Fell DB Platt RW Basso O Wilson K Kaufman JS Buckeridge DL. The relationship between 2009 pandemic H1N1 influenza during pregnancy and preterm birth: a population-based cohort study. Epidemiology. (2018) 29:107–16. 10.1097/EDE.0000000000000753 28930786

[17] Pierce M Kurinczuk JJ Spark P Brocklehurst P Knight M Ukoss. Perinatal outcomes after maternal 2009/H1N1 infection: national cohort study. BMJ. (2011) 342:d3214. 10.1136/bmj.d3214 21672992 PMC3114455

[18] Newsome K Alverson CJ Williams J McIntyre AF Fine AD Wasserman C. Outcomes of infants born to women with influenza A(H1N1)pdm09. Birth Defects Res. (2019) 111:88–95. 10.1002/bdr2.1445 30623611 PMC6771262

[19] Fonseca V Davis M Wing R Kriner P Lopez K Blair PJ. Novel influenza A (H1N1) virus infections in three pregnant women-United States, April-May 2009. MMWR Morb Mortal Wkly Rep. (2009) 58:497–500. 19444154

[20] Investigators AI Australasian Maternity Outcomes Surveillance S. Critical illness due to 2009 A/H1N1 influenza in pregnant and post-partum women: population based cohort study. BMJ. (2010) 340:c1279. 10.1136/bmj.c1279 20299694 PMC2841744

[21] Ciglenecki I Bichet M Tena J Mondesir E Bastard M Tran NT. Cholera in pregnancy: outcomes from a specialized cholera treatment unit for pregnant women in Leogane, Haiti. PLoS Negl Trop Dis. (2013) 7:e2368. 10.1371/journal.pntd.0002368 23967361 PMC3744413

[22] Liu S Sha J Yu Z Hu Y Chan TC Wang X. Avian influenza virus in pregnancy. Rev Med Virol. (2016) 26:268–84. 10.1002/rmv.1884 27187752

[23] Henwood PC Bebell LM Roshania R Wolfman V Mallow M Kalyanpur A. Ebola virus disease and pregnancy: a retrospective cohort study of patients managed at 5 Ebola treatment units in West Africa. Clin Infect Dis. (2017) 65:292–9. 10.1093/cid/cix290 28379374 PMC5850452

[24] Schuler-Faccini L Ribeiro EM Feitosa IM Horovitz DD Cavalcanti DP Pessoa A. Possible association between Zika virus infection and microcephaly-Brazil, 2015. MMWR Morb Mortal Wkly Rep. (2016) 65:59–62. 10.15585/mmwr.mm6503e2 26820244

[25] Gupta S Gupta N. Short-term pregnancy outcomes in patients chikungunya infection: an observational study. J Family Med Prim Care. (2019) 8:985–7. 10.4103/jfmpc.jfmpc_274_18 31041238 PMC6482732

[26] Barbero P Muguerza L Herraiz I Garcia Burguillo A San Juan R Forcen L. SARS-CoV-2 in pregnancy: characteristics and outcomes of hospitalized and non-hospitalized women due to COVID-19. J Matern Fetal Neonatal Med. (2022) 35:2648–54. 10.1080/14767058.2020.1793320 32689846

[27] Xu S Shao F Bao B Ma X Xu Z You J. Clinical manifestation and neonatal outcomes of pregnant patients with coronavirus disease 2019 pneumonia in Wuhan, China. Open Forum Infect Dis. (2020) 7:ofaa283. 10.1093/ofid/ofaa283 32743014 PMC7384380

[28] ACOG Committee Opinion. Influenza vaccination during pregnancy. ACOG Committee Opinion No. 732. Obstet Gynecol. (2018) 131:e109–14. 10.1097/AOG.0000000000002588 29578985

[29] Andorf S Bhattacharya S Gaudilliere B Shaw GM Stevenson DK Butte AJ. Pilot study showing a stronger H1N1 influenza vaccination response during pregnancy in women who subsequently deliver preterm. J Reprod Immunol. (2019) 132:16–20. 10.1016/j.jri.2019.02.004 30852461 PMC6456418

[30] Richards JL Hansen C Bredfeldt C Bednarczyk RA Steinhoff MC Adjaye-Gbewonyo D. Neonatal outcomes after antenatal influenza immunization during the 2009 H1N1 influenza pandemic: impact on preterm birth, birth weight, and small for gestational age birth. Clin Infect Dis. (2013) 56:1216–22. 10.1093/cid/cit045 23378281 PMC4357807

[31] Rubinstein F Micone P Bonotti A Wainer V Schwarcz A Augustovski F. Antigripal EVASRGEEyV. Influenza A/H1N1 MF59 adjuvanted vaccine in pregnant women and adverse perinatal outcomes: multicentre study. BMJ. (2013) 346:f393. 10.1136/bmj.f393 23381200 PMC3563311

[32] Kallen B Olausson PO. Vaccination against H1N1 influenza with Pandemrix((R)) during pregnancy and delivery outcome: a Swedish register study. BJOG. (2012) 119:1583–90. 10.1111/j.1471-0528.2012.03470.x 22901103

[33] WHO. Barriers of Influenza Vaccination Intention and Behavior: A Systematic Review of Influenza Vaccine Hesitancy 2005–2016. Geneva: World Health Organization (2016). 10.1371/journal.pone.0170550 PMC5268454 28125629

[34] Nakai A Saito S Unno N Kubo T Minakami H. Review of the pandemic (H1N1) 2009 among pregnant Japanese women. J Obstet Gynaecol Res. (2012) 38:757–62. 10.1111/j.1447-0756.2011.01812.x 22487092

[35] Baum U Leino T Gissler M Kilpi T Jokinen J. Perinatal survival and health after maternal influenza A(H1N1)pdm09 vaccination: a cohort study of pregnancies stratified by trimester of vaccination. Vaccine. (2015) 33:4850–7. 10.1016/j.vaccine.2015.07.061 26238723

[36] Beau AB Hurault-Delarue C Vidal S Guitard C Vayssiere C Petiot D. Pandemic A/H1N1 influenza vaccination during pregnancy: a comparative study using the EFEMERIS database. Vaccine. (2014) 32:1254–8. 10.1016/j.vaccine.2014.01.021 24486369

[37] Chambers CD Johnson DL Xu R Luo YJ Louik C Mitchell AA. Safety of the 2010-11, 2011–12, 2012–13, and 2013–14 seasonal influenza vaccines in pregnancy: birth defects, spontaneous abortion, preterm delivery, and small for gestational age infants, a study from the cohort arm of VAMPSS. Vaccine. (2016) 34:4443–9. 10.1016/j.vaccine.2016.06.054 27449682

[38] Fabiani M Bella A Rota MC Clagnan E Gallo T D'Amato M. A/H1N1 pandemic influenza vaccination: a retrospective evaluation of adverse maternal, fetal and neonatal outcomes in a cohort of pregnant women in Italy. Vaccine. (2015) 33:2240–7. 10.1016/j.vaccine.2015.03.041 25820060

[39] Getahun D Fassett MJ Peltier MR Takhar HS Shaw SF Im TM. Association between seasonal influenza vaccination with pre- and post-natal outcomes. Vaccine. (2019) 37:1785–91. 10.1016/j.vaccine.2019.02.019 30799158

[40] Sheffield JS Greer LG Rogers VL Roberts SW Lytle H McIntire DD. Effect of influenza vaccination in the first trimester of pregnancy. Obstet Gynecol. (2012) 120:532–7. 10.1097/AOG.0b013e318263a278 22914461

[41] Zerbo O Modaressi S Chan B Goddard K Lewis N Bok K. No association between influenza vaccination during pregnancy and adverse birth outcomes. Vaccine. (2017) 35:3186–90. 10.1016/j.vaccine.2017.04.074 28483192

[42] Giles ML Krishnaswamy S Macartney K Cheng A. The safety of inactivated influenza vaccines in pregnancy for birth outcomes: a systematic review. Hum Vaccin Immunother. (2019) 15:687–99. 10.1080/21645515.2018.1540807 30380986 PMC6605784

[43] Newsome K Williams J Way S Honein M Hill H Rasmussen R. Maternal and infant outcomes among severely ill pregnant and postpartum women with 2009 pandemic influenza A (H1N1)–United States, April 2009-August 2010. MMWR Morb Mortal Wkly Rep. (2011) 60:1193–6. 21900872

[44] Siston AM Rasmussen SA Honein MA Fry AM Seib K Callaghan WM. Pandemic 2009 influenza A(H1N1) virus illness among pregnant women in the United States. JAMA. (2010) 303:1517–25. 10.1001/jama.2010.479 20407061 PMC5823273

[45] Yates L Pierce M Stephens S Mill AC Spark P Kurinczuk JJ. Influenza A/H1N1v in pregnancy: an investigation of the characteristics and management of affected women and the relationship to pregnancy outcomes for mother and infant. Health Technol Assess. (2010) 14:109–82. 10.3310/hta14340-02 20630123

[46] Graner S Svensson T Beau AB Damase-Michel C Engeland A Furu K. Neuraminidase inhibitors during pregnancy and risk of adverse neonatal outcomes and congenital malformations: population based European register study. BMJ. (2017) 356:j629. 10.1136/bmj.j629 28246106 PMC5421412

[47] Parsons JE Newby KV French DP Bailey E Inglis N. The development of a digital intervention to increase influenza vaccination amongst pregnant women. Digit Health. (2021) 7:20552076211012128. 10.1177/20552076211012128 33996140 PMC8076770

[48] Jamieson DJ Uyeki TM Callaghan WM Meaney-Delman D Rasmussen SA. What obstetrician-gynecologists should know about Ebola: a perspective from the centers for disease control and prevention. Obstet Gynecol. (2014) 124:1005–10. 10.1097/AOG.0000000000000533 25203368

[49] Bebell LM Oduyebo T Riley LE. Ebola virus disease and pregnancy: a review of the current knowledge of Ebola virus pathogenesis, maternal, and neonatal outcomes. Birth Defects Res. (2017) 109:353–62. 10.1002/bdra.23558 28398679 PMC5407292

[50] Mupapa K Mukundu W Bwaka MA Kipasa M De Roo A Kuvula K. Ebola hemorrhagic fever and pregnancy. J Infect Dis. (1999) 179 Suppl 1:S11–2. 10.1086/514289 9988157

[51] Strom BL Buyse M Hughes J Knoppers BM. Data sharing, year 1—access to data from industry-sponsored clinical trials. N Engl J Med. (2014) 371:2052–4. 10.1056/NEJMp1411794 25317745

[52] Schieffelin JS Shaffer JG Goba A Gbakie M Gire SK Colubri A. Clinical illness and outcomes in patients with Ebola in Sierra Leone. N Engl J Med. (2014) 371:2092–100. 10.1056/NEJMoa1411680 25353969 PMC4318555

[53] Caluwaerts S Fautsch T Lagrou D Moreau M Modet Camara A Gunther S. Dilemmas in managing pregnant women With Ebola: 2 case reports. CLin Infect Dis. (2016) 62:903–5. 10.1093/cid/civ1024 26679622 PMC4787604

[54] Bower H Grass JE Veltus E Brault A Campbell S Basile AJ. Delivery of an Ebola virus-positive stillborn infant in a rural community health center, Sierra Leone, 2015. Am J Trop Med Hyg. (2016) 94:417–9. 10.4269/ajtmh.15-0619 26556830 PMC4751940

[55] Akerlund E Prescott J Tampellini L. Shedding of Ebola virus in an asymptomatic pregnant woman. New England J Med. (2015) 372:2467–9. 10.1056/NEJMc1503275 26083224

[56] Baggi FM Taybi A Kurth A Van Herp M Di Caro A Wolfel R. Management of pregnant women infected with Ebola virus in a treatment centre in Guinea, June 2014. Euro Surveill. (2014) 19:20983. 10.2807/1560-7917.ES2014.19.49.20983 25523968

[57] Dornemann J Burzio C Ronsse A Sprecher A De Clerck H Van Herp M. First newborn baby to receive experimental therapies survives ebola virus disease. J Infect Dis. (2017) 215:171–4. 10.1093/infdis/jiw493 28073857 PMC5583641

[58] Henao-Restrepo AM Camacho A Longini IM Watson CH Edmunds WJ Egger M. Efficacy and effectiveness of an rVSV-vectored vaccine in preventing Ebola virus disease: final results from the Guinea ring vaccination, open-label, cluster-randomised trial (Ebola Ca Suffit!). Lancet. (2017) 389:505–18. 10.1016/S0140-6736(16)32621-6 28017403 PMC5364328

[59] WHO. Guidelines for the Management of Pregnant and Breastfeeding Women in the Context of Ebola Virus Disease. Geneva: World Health Organisation (2020). 32091684

[60] Kraus N Condon SB. Measles (Rubeola): a case of vaccine hesitancy and pregnancy. J Midwifery Womens Health. (2021) 66:391–6. 10.1111/jmwh.13223 34022106

[61] Allam MF. New measles vaccination schedules in the European countries? J Prev Med Hyg. (2014) 55:33–4. 25916031 PMC4718332

[62] Ragusa R Platania A Cuccia M Zappala G Giorgianni G D'Agati P. Measles and pregnancy: immunity and immunization-what can be learned from observing complications during an epidemic year. J Pregnancy. (2020) 2020:6532868. 10.1155/2020/6532868 32802510 PMC7416282

[63] Anselem O Tsatsaris V Lopez E Krivine A Le Ray C Loulergue P. Measles and pregnancy. Presse Med. (2011) 40:1001–7. 10.1016/j.lpm.2011.07.002 21885237

[64] Mwangome M Ngari M Bwahere P Kabore P McGrath M Kerac M. Anthropometry at birth and at age of routine vaccination to predict mortality in the first year of life: a birth cohort study in BukinaFaso. PLoS ONE. (2019) 14:e0213523. 10.1371/journal.pone.0213523 30921335 PMC6438502

[65] Gonik B Puder KS Gonik N Kruger M. Seroprevalence of Bordetella pertussis antibodies in mothers and their newborn infants. Infect Dis Obstet Gynecol. (2005) 13:59–61. 10.1080/10647440500068289 16011994 PMC1784563

[66] Agrawal A Singh S Kolhapure S Kandeil W Pai R Singhal T. Neonatal pertussis, an under-recognized health burden and rationale for maternal immunization: a systematic review of South and South-East Asian countries. Infect Dis Ther. (2019) 8:139–53. 10.1007/s40121-019-0245-2 31054089 PMC6522626

[67] Moro PL McNeil MM Sukumaran L Broder KR. The centers for disease control and prevention's public health response to monitoring Tdap safety in pregnant women in the United States. Hum Vaccin Immunother. (2015) 11:2872–9. 10.1080/21645515.2015.1072664 26378718 PMC5054779

[68] Abu-Raya B Maertens K Edwards KM Omer SB Englund JA Flanagan KL. Global perspectives on immunization during pregnancy and priorities for future research and development: an international consensus statement. Front Immunol. (2020) 11:1282. 10.3389/fimmu.2020.01282 32670282 PMC7326941

[69] Furuta M Sin J Ng ESW Wang K. Efficacy and safety of pertussis vaccination for pregnant women - a systematic review of randomised controlled trials and observational studies. BMC Pregnancy Childbirth. (2017) 17:390. 10.1186/s12884-017-1559-2 29166874 PMC5700667

[70] Kharbanda EO Vazquez-Benitez G Lipkind HS Klein NP Cheetham TC Naleway A. Evaluation of the association of maternal pertussis vaccination with obstetric events and birth outcomes. JAMA. (2014) 312:1897–904. 10.1001/jama.2014.14825 25387187 PMC6599584

[71] Lowe R Barcellos C Brasil P Cruz OG Honorio NA Kuper H. The Zika virus epidemic in brazil: from discovery to future implications. Int J Environ Res Public Health. (2018) 15:96. 10.3390/ijerph15010096 29315224 PMC5800195

[72] Martins MM Alves da. Cunha AJL, Robaina JR, Raymundo CE, Barbosa AP, Medronho RA. Fetal, neonatal, and infant outcomes associated with maternal Zika virus infection during pregnancy: A systematic review and meta-analysis. PLoS ONE. (2021) 16:e0246643. 10.1371/journal.pone.0246643 33606729 PMC7894820

[73] WHO. WHO Guidelines for the Prevention of Sexual transmission of Zika Virus: Executive Summary. Geneva: World Health Organisation (2019). 32609457

[74] Quanquin N Wang L Cheng G. Potential for treatment and a Zika virus vaccine. Curr Opin Pediatr. (2017) 29:114–21. 10.1097/MOP.0000000000000441 27906864 PMC5963532

[75] Mohd HA Al-Tawfiq JA Memish ZA. Middle East respiratory syndrome coronavirus (MERS-CoV) origin and animal reservoir. Virol J. (2016) 13:87. 10.1186/s12985-016-0544-0 27255185 PMC4891877

[76] Payne DC Iblan I Alqasrawi S Al Nsour M Rha B Tohme RA. Stillbirth during infection with Middle East respiratory syndrome coronavirus. J Infect Dis. (2014) 209:1870–2. 10.1093/infdis/jiu068 24474813 PMC4618552

[77] Rabaan AA Al-Ahmed SH Sah R Alqumber MA Haque S Patel SK. MERS-CoV: epidemiology, molecular dynamics, therapeutics, and future challenges. Ann Clin Microbiol Antimicrob. (2021) 20:8. 10.1186/s12941-020-00414-7 33461573 PMC7812981

[78] Ahmadzadeh J Mobaraki K Mousavi SJ Aghazadeh-Attari J Mirza-Aghazadeh-Attari M Mohebbi I. The risk factors associated with MERS-CoV patient fatality: a global survey. Diagn Microbiol Infect Dis. (2020) 96:114876. 10.1016/j.diagmicrobio.2019.114876 31959375 PMC7126953

[79] Alserehi H Wali G Alshukairi A Alraddadi B. Impact of Middle East Respiratory Syndrome coronavirus (MERS-CoV) on pregnancy and perinatal outcome. BMC Infect Dis. (2016) 16:105. 10.1186/s12879-016-1437-y 26936356 PMC4776369

[80] Wastnedge EAN Reynolds RM van Boeckel SR Stock SJ Denison FC Maybin JA. Pregnancy and COVID-19. Physiol Rev. (2021) 101:303–18. 10.1152/physrev.00024.2020 32969772 PMC7686875

[81] Yoon SH Kang JM Ahn JG. Clinical outcomes of 201 neonates born to mothers with COVID-19: a systematic review. Eur Rev Med Pharmacol Sci. (2020) 24:7804–15. 10.26355/eurrev_202007_22285 32744708

[82] Chi H Chiu NC Tai YL Chang HY Lin CH Sung YH. Clinical features of neonates born to mothers with coronavirus disease-2019: a systematic review of 105 neonates. J Microbiol Immunol Infect. (2021) 54:69–76. 10.1016/j.jmii.2020.07.024 32847748 PMC7427525

[83] Zhu H Wang L Fang C Peng S Zhang L Chang G. Clinical analysis of 10 neonates born to mothers with 2019-nCoV pneumonia. Transl Pediatr. (2020) 9:51–60. 10.21037/tp.2020.02.06 32154135 PMC7036645

[84] Chen H Guo J Wang C Luo F Yu X Zhang W. Clinical characteristics and intrauterine vertical transmission potential of COVID-19 infection in nine pregnant women: a retrospective review of medical records. Lancet. (2020) 395:809–15. 10.1016/S0140-6736(20)30360-3 32151335 PMC7159281

[85] Aabakke AJM Krebs L Petersen TG Kjeldsen FS Corn G Wojdemann K. SARS-CoV-2 infection in pregnancy in Denmark-characteristics and outcomes after confirmed infection in pregnancy: a nationwide, prospective, population-based cohort study. Acta Obstet Gynecol Scand. (2021) 100:2097–110. 10.1111/aogs.14252 34467518 PMC8652723

[86] Stephansson O Pasternak B Ahlberg M Hervius Askling H Aronsson B Appelqvist E. SARS-CoV-2 and pregnancy outcomes under universal and non-universal testing in Sweden: register-based nationwide cohort study. BJOG. (2022) 129:282–90. 10.1111/1471-0528.16990 34706148 PMC8652549

[87] Shafran N Shafran I Ben-Zvi H Sofer S Sheena L Krause I. Secondary bacterial infection in COVID-19 patients is a stronger predictor for death compared to influenza patients. Sci Rep. (2021) 11:12703. 10.1038/s41598-021-92220-0 34135459 PMC8209102

[88] Collins S Ramsay M Slack MP Campbell H Flynn S Litt D. Risk of invasive Haemophilus influenzae infection during pregnancy and association with adverse fetal outcomes. JAMA. (2014) 311:1125–32. 10.1001/jama.2014.1878 24643602

[89] Kc A Gurung R Kinney MV Sunny AK Moinuddin M Basnet O. Effect of the COVID-19 pandemic response on intrapartum care, stillbirth, and neonatal mortality outcomes in Nepal: a prospective observational study. Lancet Glob Health. (2020) 8:e1273–81. 10.1016/S2214-109X(20)30345-4 32791117 PMC7417164

[90] Alshaikh B Cheung PY Soliman N Brundler MA Yusuf K. Impact of lockdown measures during COVID-19 pandemic on pregnancy and preterm birth. Am J Perinatol. (2022) 39:329–36. 10.1055/s-0041-1739357 34775579

[91] Arun Babu T Sharmila V Vishnu Bhat B. Curious scenario of changes in incidence of preterm births during COVID-19 pandemic. Pointers for future research? Eur J Obstet Gynecol Reprod Biol. (2020) 253:333–4. 10.1016/j.ejogrb.2020.08.055 32891485 PMC7456271

[92] Hedermann G Hedley PL Baekvad-Hansen M Hjalgrim H Rostgaard K Poorisrisak P. Danish premature birth rates during the COVID-19 lockdown. Arch Dis Child Fetal Neonatal Ed. (2021) 106:93–5. 10.1136/archdischild-2020-319990 32788391 PMC7421710

[93] Hedley PL Hedermann G Hagen CM Baekvad-Hansen M Hjalgrim H Rostgaard K. Preterm birth, stillbirth and early neonatal mortality during the Danish COVID-19 lockdown. Eur J Pediatr. (2022) 181:1175–84. 10.1007/s00431-021-04297-4 34783897 PMC8593096

[94] Philip RK Purtill H Reidy E Daly M Imcha M McGrath D. Reduction in preterm births during the COVID-19 lockdown in Ireland: a natural experiment allowing analysis of data from the prior two decades. BMJ Global Health. (2020). 10.1101/2020.06.03.20121442 PMC7528371 32999054

[95] Preston E. During coronavirus lockdowns, some doctors wondered: where are the preemies? The New York Times. (2020).

[96] Li M Yin H Jin Z Zhang H Leng B Luo Y. Impact of Wuhan lockdown on the indications of cesarean delivery and newborn weights during the epidemic period of COVID-19. PLoS One. (2020) 15:e0237420. 10.1371/journal.pone.0237420 32790709 PMC7425855

[97] Emanoil AR Stochino Loi E Feki A Ben Ali N. Focusing treatment on pregnant women with COVID disease. Front Glob Womens Health. (2021) 2:590945. 10.3389/fgwh.2021.590945 34816175 PMC8593971

[98] Wilson RJ Paterson P Jarrett C Larson HJ. Understanding factors influencing vaccination acceptance during pregnancy globally: a literature review. Vaccine. (2015) 33:6420–9. 10.1016/j.vaccine.2015.08.046 26320417

[99] Skjefte M Ngirbabul M Akeju O Escudero D Hernandez-Diaz S Wyszynski DF. COVID-19 vaccine acceptance among pregnant women and mothers of young children: results of a survey in 16 countries. Eur J Epidemiol. (2021) 36:197–211. 10.1007/s10654-021-00728-6 33649879 PMC7920402

[100] Nordman H Jaaskelainen J Voutilainen R. Birth size as a determinant of cardiometabolic risk factors in children. Horm Res Paediatr. (2020) 93:144–53. 10.1159/000509932 32846418

[101] Wardlaw T You D Hug L Amouzou A Newby H UNICEF. Report: enormous progress in child survival but greater focus on newborns urgently needed. Reprod Health. (2014) 11:82. 10.1186/1742-4755-11-82 25480451 PMC4320591

[102] Nations U,. Ensure Healthy Lives Promote Well-being for all at All Ages. (2022). Available online at: https://unstats.un.org/sdgs/report/2021/goal-03/ (accessed August 11, 2022).

[103] Malange VNE Hedermann G Lausten-Thomsen U Hoffmann S Voldstedlund M Aabakke A. The Perinatal Health Challenges of Emerging and Re-Emerging Infectious Diseases: A Narrative Review. 10.2139/ssrn.4198174 PMC9850110 36684933

