# Supplementary material for: The perinatal health challenges of emerging and re-emerging infectious diseases: A narrative review
Source: Front Public Health. 2023 Jan 5;10:1039779. doi: 10.3389/fpubh.2022.1039779 (PMC9850110; doi:10.3389/fpubh.2022.1039779)
Supplement: Supplementary file 1 [file Data_Sheet_1.docx]

Supplementary Material

## Supplementary Figures


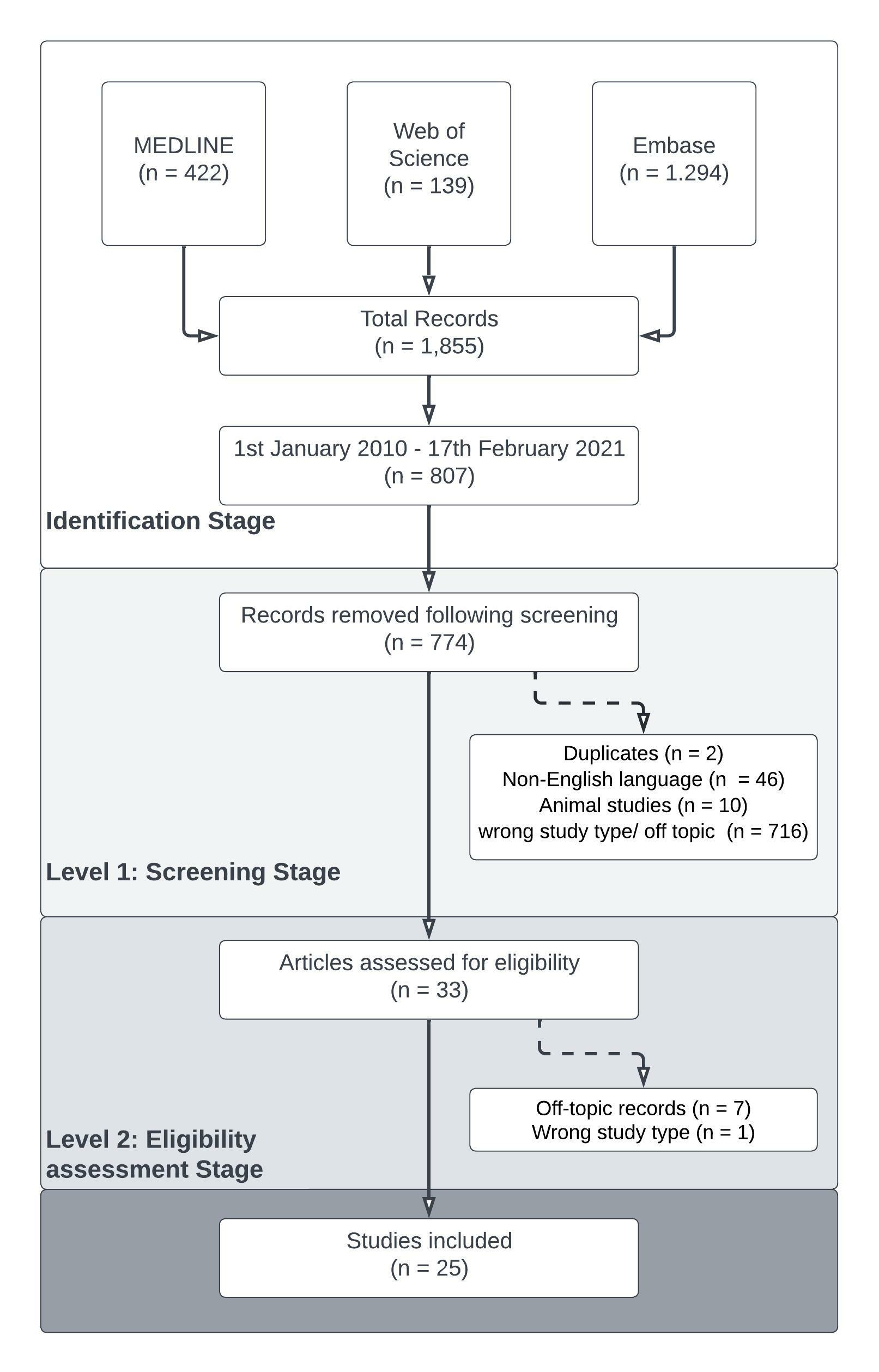


**Supplementary Figure 1.** Prisma flow chart of literature review process for studies examining perinatal health outcomes during infectious disease outbreaks.

## Supplementary Tables

**Supplementary Table 1:** List of Search terms used for each P. E. O. concept. The concepts were combined in the search using an AND operator. Search terms presented in a Medline format, the format was adapted to each database searched.

| **Population** | Neonat* OR Newborn* |
| --- | --- |
| **Exposure** | Disease adj1 outbreaks, infectious disease* OR Communicable Disease Control OR pandemics OR epidemics |
| **Outcome** | "aborted f?etus" OR apgar OR "birth defect" OR "congenital abnormalit*" OR "congenital defect" OR "congenital infection" OR "congenital malformation" OR "f?etal anomal*" OR "f?etal death" OR "f?etal malformation" OR "f?etal mortality" OR "infant death" OR "infant mortality" OR "low birth weight" OR "maternal death" OR "maternal mortality" OR "microcephaly" OR "miscarriage" OR "neonatal death" OR "neonatal mortality" OR "perinatal death" OR "perinatal mortality" OR "premature birth" OR "premature labo?r" OR "preterm birth" OR "small for gestational age" OR "spontaneous abortion” OR stillbirth |

**Supplementary Table 2:** Characteristics of the studies identified using the search strategy described in Supplementary Table 1 and Supplementary Figure 1.

| **Reference** | **Country** | **Study Design** | **Study Period** | **Exposure of interest** | **Exposed** | **Non-exposed** | **Main outcome measures** |
| --- | --- | --- | --- | --- | --- | --- | --- |
| Yates et al, 2010 (1) | United Kingdom | Cohort study | September 7, 2009 - January 29, 2010 | H1N1 influenza virus | 1,428 | 570 | adverse pregnancy outcomes (e.g. PTB), adverse drug and vaccine effects |
| CDC, 2011 (2) | United States | Cross-sectional study | April 15, 2009 - August 10, 2010 | H1N1 influenza virus | 347 |  | underlying conditions, treatment type and outcome |
| Fell et al, 2012 (3) | Canada | Cohort study | November 2, 2009 - April 30, 2010 | H1N1 vaccination during pregnancy | 23,340 | 32,230 | PTB, SGA, 5-min Apgar, and fetal death |
| Kallen & Olausson, 2012 (4) | Sweden | Cohort study | 2009-2010 | Pandemrix | 18,612 | 220,212 | stillbirth, PTB, LBW, SGA, congenital malformations |
| Pasternak et al, 2012 (5) | Denmark | Cohort study | November 2, 2009 - September 30, 2010 | Pandemrix | 7,062 | 47,523 | fetal death (stillbirth & abortion) |
| Pasternak et al, 2012 (6) | Denmark | Cohort study | November 2, 2009 - September 30, 2010 | Pandemrix | 6,989 | 46,443 | major birth defects, PTB & SGA |
| Ludvigsson et al, 2013 (7) | Sweden | Cohort study | February, 2009 - January, 2010 | Pandemrix | 13,297 | 7,790 | LBW, PTB, SGA, 5-min Apgar, and caesarean section |
| Richards et al, 2013 (8) | United States | Cohort study | April 26, 2009 - April 17, 2010 | 2009 H1N1 influenza vaccine or seasonal trivalent inactivated vaccine [TIV] | 1,125 | 1,581 | PTB, birth weight, LBW, & SGA |
| Rubinstein et al, 2013 (9) | Argentina | Cross-sectional study | September, 2010 - May, 2011 | MF59-adjuvanted A/H1N1 vaccine | 7,293 | 23,195 | LBW, PTB, fetal death, & neonatal death |
| Beau et al, 2014 (10) | France | Cohort study | October 21, 2009 - November 30, 2010 | A/H1N1 vaccination | 1,645 | 3,290 | pregnancy loss, PTB, SGA, & neonatal pathology |
| Cleary et al, 2014 (11) | Ireland | Cohort study | December, 2008-2009 - September, 2009-2010 | Celvapan or Pandemrix | 2,996 | 3,898 | PTB, SGA, NICU admission, congenital anomalies, & perinatal death |
| Kharbanda et al, 2014 (12) | United States | Cohort study | January 1, 2010 - November 15, 2012 | tetanus toxoid, reduced diphtheria toxoid, and acellular pertussis vaccine (Tdap) | 26,229 | 97,265 | SGA, chorioamnionitis, PTB, hypertensive disorders of pregnancy |
| Baum et al, 2015 (13) | Finland | Cohort study | November 1, 2009 - delivery | Pandemrix | 34,241 | 9,363 | stillbirth, early neonatal death, PTB, LBW, & SGA |
| Fabiani et al, 2015 (14) | Italy | Cohort study | October 15, 2009 - September 30, 2010 | MF59-adjuvanted A/H1N1 vaccine | 2,048 | 100,029 | maternal hospital admissions, stillbirth, PTB, LBW, 5-min Apgar, congenital malformations, & infant hospital admissions (≤ 6 months) |
| Millar & Sanz, 2015 (15) | United States | Cohort study | January 1, 2010 - November 15, 2012 | tetanus toxoid, reduced diphtheria toxoid, and acellular pertussis vaccine (Tdap) | 26,195 | 97,299 | hypertensive disorder in pregnancy, chorioamnionitis, PTB, SGA |
| Chambers et al, 2016 (16) | United States & Canada | Cohort study | 2010 - 2014 | seasonal influenza vaccine | 1,263 | 467 | Birth outcome, gestational age at outcome, mode of delivery, sex and number of infants, birth weight, length, and head circumference and major birth defects detected up through the first year of life. |
| Arriola et al, 2017 (17) | Nicaragua | Cohort study | July 21, - December 4, 2014 | Trivalent inactivated influenza vaccine, southern hemisphere formulation | 1,789 | 1,479 | SGA, PTB, & LBW |
| Zerbo et al, 2017 (18) | United States | Cohort study | January 1, 2010 - December 31, 2015 | Maternal influenza vaccination | 64,748 | 81,119 | PTB, SGA, LGA, NICU admission, Respiratory distress syndrome, LBW, 5-min Apgar |
| Getahun et al, 2019 (19) | United States | Cohort study | January 1, 2008 - December 31, 2016 | Seasonal influenza vaccination | 130,996 | 116,040 | influenza, PTB, PROM, chorioamnionitis, SGA, preeclampsia, placental abruption, stillbirth, NICU admission and length of stay |
| McHugh et al, 2019 (20) | Australia | Cohort study | January 1, 1994 - December 31,2014 | influenza or pertussis infant hospitalisations | 15,346 | 35,951 | PTB, stillbirth, LBW, & SGA |
| Newsome et al, 2019 (21) | United States | Case-control study | April 1, - August 21, 2009 | H1N1 influenza virus | 490 | 1,451 | PTB, LBW, 5-min Apgar |
| Rolfes et al, 2019 (22) | Lao PDR | Cohort study | April 2, 2014 ‐ February 27, 2015 | Influenza vaccination | 2,142 | 2,692 | PTB, SGA |
| Li et al, 2020 (23) | China | Cohort study | January 23, 2019 - March 24, 2020 | Lockdown restrictions | 3,432 | 7,159 | delivery mode, birth weight |
| Woodworth et al, 2020 (24) | United States | Cross-sectional study | March 29, - October 14, 2020 | SARS-CoV2 | 4,442 |  | PTB, NICU admission, neonatal SARS-CoV2 infection |
| Dawood et al, 2021 (25) | India, Peru, Thailand | Cohort study | March 13, 2017 - August 3, 2018 | Influenza virus | 4,791 | 6,035 | Acute respiratory illness (ARI), ARI-associated hospitalisation, febrile ARI, rtPCR confirmed influenza, PTB, late pregnancy loss, SGA, & birth weight |

References

1. Yates L, Pierce M, Stephens S, Mill AC, Spark P, Kurinczuk JJ, Valappil M, Brocklehurst P, Thomas SH, Knight M. Influenza A/H1N1v in pregnancy: an investigation of the characteristics and management of affected women and the relationship to pregnancy outcomes for mother and infant. Health Technol Assess. 2010 Jul;14(34):109-82. doi:10.3310/hta14340-02. Cited in: Pubmed; PMID 20630123.

2. Centers for Disease C, Prevention. Maternal and infant outcomes among severely ill pregnant and postpartum women with 2009 pandemic influenza A (H1N1)--United States, April 2009-August 2010. MMWR Morbidity and mortality weekly report. 2011;60(35):1193-6.

3. Fell DB, Sprague AE, Liu N, Yasseen AS, 3rd, Wen SW, Smith G, Walker MC, Better Outcomes R, Network O. H1N1 influenza vaccination during pregnancy and fetal and neonatal outcomes. Am J Public Health. 2012 Jun;102(6):e33-40. doi:10.2105/AJPH.2011.300606. Cited in: Pubmed; PMID 22515877.

4. Kallen B, Olausson PO. Vaccination against H1N1 influenza with Pandemrix((R)) during pregnancy and delivery outcome: a Swedish register study. BJOG. 2012 Dec;119(13):1583-90. doi:10.1111/j.1471-0528.2012.03470.x. Cited in: Pubmed; PMID 22901103.

5. Pasternak B, Svanstrom H, Molgaard-Nielsen D, Krause TG, Emborg HD, Melbye M, Hviid A. Vaccination against pandemic A/H1N1 2009 influenza in pregnancy and risk of fetal death: cohort study in Denmark. BMJ. 2012 May 2;344:e2794. doi:10.1136/bmj.e2794. Cited in: Pubmed; PMID 22551713.

6. Pasternak B, Svanstrom H, Molgaard-Nielsen D, Krause TG, Emborg HD, Melbye M, Hviid A. Risk of adverse fetal outcomes following administration of a pandemic influenza A(H1N1) vaccine during pregnancy. JAMA. 2012 Jul 11;308(2):165-74. doi:10.1001/jama.2012.6131. Cited in: Pubmed; PMID 22782418.

7. Ludvigsson JF, Zugna D, Cnattingius S, Richiardi L, Ekbom A, Ortqvist A, Persson I, Stephansson O. Influenza H1N1 vaccination and adverse pregnancy outcome. Eur J Epidemiol. 2013 Jul;28(7):579-88. doi:10.1007/s10654-013-9813-z. Cited in: Pubmed; PMID 23715672.

8. Richards JL, Hansen C, Bredfeldt C, Bednarczyk RA, Steinhoff MC, Adjaye-Gbewonyo D, Ault K, Gallagher M, Orenstein W, Davis RL, Omer SB. Neonatal outcomes after antenatal influenza immunization during the 2009 H1N1 influenza pandemic: impact on preterm birth, birth weight, and small for gestational age birth. Clin Infect Dis. 2013 May;56(9):1216-22. doi:10.1093/cid/cit045. Cited in: Pubmed; PMID 23378281.

9. Rubinstein F, Micone P, Bonotti A, Wainer V, Schwarcz A, Augustovski F, Pichon Riviere A, Karolinski A, Antigripal EVASRGEEyV. Influenza A/H1N1 MF59 adjuvanted vaccine in pregnant women and adverse perinatal outcomes: multicentre study. BMJ. 2013 Feb 4;346:f393. doi:10.1136/bmj.f393. Cited in: Pubmed; PMID 23381200.

10. Beau AB, Hurault-Delarue C, Vidal S, Guitard C, Vayssiere C, Petiot D, Montastruc JL, Damase-Michel C, Lacroix I. Pandemic A/H1N1 influenza vaccination during pregnancy: a comparative study using the EFEMERIS database. Vaccine. 2014 Mar 5;32(11):1254-8. doi:10.1016/j.vaccine.2014.01.021. Cited in: Pubmed; PMID 24486369.

11. Cleary BJ, Rice U, Eogan M, Metwally N, McAuliffe F. 2009 A/H1N1 influenza vaccination in pregnancy: uptake and pregnancy outcomes - a historical cohort study. Eur J Obstet Gynecol Reprod Biol. 2014 Jul;178:163-8. doi:10.1016/j.ejogrb.2014.04.015. Cited in: Pubmed; PMID 24793932.

12. Kharbanda EO, Vazquez-Benitez G, Lipkind HS, Klein NP, Cheetham TC, Naleway A, Omer SB, Hambidge SJ, Lee GM, Jackson ML, McCarthy NL, DeStefano F, Nordin JD. Evaluation of the association of maternal pertussis vaccination with obstetric events and birth outcomes. JAMA. 2014 Nov 12;312(18):1897-904. doi:10.1001/jama.2014.14825. Cited in: Pubmed; PMID 25387187.

13. Baum U, Leino T, Gissler M, Kilpi T, Jokinen J. Perinatal survival and health after maternal influenza A(H1N1)pdm09 vaccination: A cohort study of pregnancies stratified by trimester of vaccination. Vaccine. 2015 Sep 11;33(38):4850-7. doi:10.1016/j.vaccine.2015.07.061. Cited in: Pubmed; PMID 26238723.

14. Fabiani M, Bella A, Rota MC, Clagnan E, Gallo T, D'Amato M, Pezzotti P, Ferrara L, Demicheli V, Martinelli D, Prato R, Rizzo C. A/H1N1 pandemic influenza vaccination: A retrospective evaluation of adverse maternal, fetal and neonatal outcomes in a cohort of pregnant women in Italy. Vaccine. 2015 May 5;33(19):2240-2247. doi:10.1016/j.vaccine.2015.03.041. Cited in: Pubmed; PMID 25820060.

15. Millar MR, Sanz MG. The administration of pertussis vaccine to pregnant women was associated with a small increased risk of chorioamnionitis, but not an increased risk of hypertensive disorders or preterm birth. Evid Based Med. 2015 Apr;20(2):73. doi:10.1136/ebmed-2014-110149. Cited in: Pubmed; PMID 25686618.

16. Chambers CD, Johnson DL, Xu R, Luo YJ, Louik C, Mitchell AA, Schatz M, Jones KL, Group OCR. Safety of the 2010-11, 2011-12, 2012-13, and 2013-14 seasonal influenza vaccines in pregnancy: Birth defects, spontaneous abortion, preterm delivery, and small for gestational age infants, a study from the cohort arm of VAMPSS. Vaccine. 2016 Aug 17;34(37):4443-9. doi:10.1016/j.vaccine.2016.06.054. Cited in: Pubmed; PMID 27449682.

17. Arriola CS, Vasconez N, Thompson MG, Olsen SJ, Moen AC, Bresee J, Ropero AM. Association of influenza vaccination during pregnancy with birth outcomes in Nicaragua. Vaccine. 2017 May 25;35(23):3056-3063. doi:10.1016/j.vaccine.2017.04.045. Cited in: Pubmed; PMID 28465095.

18. Zerbo O, Modaressi S, Chan B, Goddard K, Lewis N, Bok K, Fireman B, Klein NP, Baxter R. No association between influenza vaccination during pregnancy and adverse birth outcomes. Vaccine. 2017 May 31;35(24):3186-3190. doi:10.1016/j.vaccine.2017.04.074. Cited in: Pubmed; PMID 28483192.

19. Getahun D, Fassett MJ, Peltier MR, Takhar HS, Shaw SF, Im TM, Chiu VY, Jacobsen SJ. Association between seasonal influenza vaccination with pre- and postnatal outcomes. Vaccine. 2019 Mar 22;37(13):1785-1791. doi:10.1016/j.vaccine.2019.02.019. Cited in: Pubmed; PMID 30799158.

20. McHugh L, Andrews RM, Leckning B, Snelling T, Binks MJ. Baseline incidence of adverse birth outcomes and infant influenza and pertussis hospitalisations prior to the introduction of influenza and pertussis vaccination in pregnancy: a data linkage study of 78 382 mother-infant pairs, Northern Territory, Australia, 1994-2015. Epidemiol Infect. 2019 Jan;147:e233. doi:10.1017/S0950268819001171. Cited in: Pubmed; PMID 31364572.

21. Newsome K, Alverson CJ, Williams J, McIntyre AF, Fine AD, Wasserman C, Lofy KH, Acosta M, Louie JK, Jones-Vessey K, Stanfield V, Yeung A, Rasmussen SA. Outcomes of infants born to women with influenza A(H1N1)pdm09. Birth Defects Res. 2019 Jan 15;111(2):88-95. doi:10.1002/bdr2.1445. Cited in: Pubmed; PMID 30623611.

22. Rolfes MA, Vonglokham P, Khanthamaly V, Chitry B, Pholsena V, Chitranondh V, Mirza SA, Moen A, Bresee JS, Xeuatvongsa A, Olsen SJ. Measurement of birth outcomes in analyses of the impact of maternal influenza vaccination. Influenza Other Respir Viruses. 2019 Nov;13(6):547-555. doi:10.1111/irv.12673. Cited in: Pubmed; PMID 31424627.

23. Li M, Yin H, Jin Z, Zhang H, Leng B, Luo Y, Zhao Y. Impact of Wuhan lockdown on the indications of cesarean delivery and newborn weights during the epidemic period of COVID-19. PLoS One. 2020;15(8):e0237420. doi:10.1371/journal.pone.0237420. Cited in: Pubmed; PMID 32790709.

24. Woodworth KR, Olsen EO, Neelam V, Lewis EL, Galang RR, Oduyebo T, Aveni K, Yazdy MM, Harvey E, Longcore ND, Barton J, Fussman C, Siebman S, Lush M, Patrick PH, Halai UA, Valencia-Prado M, Orkis L, Sowunmi S, Schlosser L, Khuwaja S, Read JS, Hall AJ, Meaney-Delman D, Ellington SR, Gilboa SM, Tong VT, Pregnancy CC-R, Infant Linked Outcomes T, Pregnancy C-, Infant Linked Outcomes T. Birth and Infant Outcomes Following Laboratory-Confirmed SARS-CoV-2 Infection in Pregnancy - SET-NET, 16 Jurisdictions, March 29-October 14, 2020 [Delaney, Augustina. CDC.]. MMWR Morb Mortal Wkly Rep. 2020 Nov 6;69(44):1635-1640. doi:10.15585/mmwr.mm6944e2. Cited in: Pubmed; PMID 33151917.

25. Dawood FS, Kittikraisak W, Patel A, Rentz Hunt D, Suntarattiwong P, Wesley MG, Thompson MG, Soto G, Mundhada S, Arriola CS, Azziz-Baumgartner E, Brummer T, Cabrera S, Chang HH, Deshmukh M, Ellison D, Florian R, Gonzales O, Kurhe K, Kaoiean S, Rawangban B, Lindstrom S, Llajaruna E, Mott JA, Saha S, Prakash A, Mohanty S, Sinthuwattanawibool C, Tinoco Y. Incidence of influenza during pregnancy and association with pregnancy and perinatal outcomes in three middle-income countries: a multisite prospective longitudinal cohort study. Lancet Infect Dis. 2021 Jan;21(1):97-106. doi:10.1016/S1473-3099(20)30592-2. Cited in: Pubmed; PMID 33129424.
